# Migraine and functional connectivity: an innovative pathophysiological perspective.

**DOI:** 10.1186/1129-2377-16-S1-A10

**Published:** 2015-09-28

**Authors:** Marina de Tommaso, Katia Ricci, Eleonora Vecchio, Daniele Marinazzo, Gabriele Trotta, Sebastiano Stramaglia

**Affiliations:** Basic Medical Neuroscience and Sensory System Department, Bari Aldo Moro University, Bari, Italy; Department of Data Analysis Faculty of Psychological and Pedagogical Sciences 1, Gent University, Ghent, Belgium; Physic Department, Bari Aldo Moro University, Bari, Italy

Migraine is a chronic disorder of neuro-vascular origin, characterized by abnormal neuronal excitability and altered processing of multimodal stimuli. Methods able to detect subtle changes of EEG rhythms under painful stimulation may improve the knowledge of mechanisms of pain processing in normal subjects and patients with chronic pain syndromes. The study of the dynamical relationships between signals recorded at different scalp locations can help to confirm and formulate hypotheses on the physiological mechanisms related to stimuli processing. Correlations, spectral coherence and phase synchronization, which allow to understand the extent to which two variables are statistically connected or shared, influenced by a third variable, together with analyses of the directionality of these dynamical interactions, may potentially contribute to understanding the mechanism of pain processing in migraine. Functional and effective connectivity in terms of synchronization and information transfer were able to reveal differences in visual reactivity between migraine patients and controls, so these methods may presumably outline a different way to process nociceptive laser stimuli in migraine, giving further knowledge on how the cortex changes its inter-connections under painful inputs.

Thirty-one migraine without aura outpatients (MwoA) were evaluated and compared to 19 controls (CONT). The right hand was stimulated by means of 30 consecutive CO2 laser stimuli. EEG signal was examined by means of Morlet wavelet, synchronization entropy and Granger causality, and the statistic results embedded into a scalp model.

The vertex complex of averaged laser evoked responses (LEPs) showed reduced habituation compared to controls. In the pre-stimulus phase enhanced synchronization entropy in the 0, 5-30 Hz range was present in MwoA and CONT between the bilateral temporal parietal and the frontal regions around the midline. Migraine patients showed an anticipation of EEG changes preceding the painful stimulation compared to controls. In the post-stimulus phase, the same cortical areas were more connected in MwoA vs CONT. In the totality of patients and controls, the habituation index was negatively correlated with the Granger Causality scores.

A different pattern of cortical activation after painful stimulation was present in migraine. The increase in cortical connections during repetitive painful stimulation may subtend the phenomenon of LEPs reduced habituation.

Brain network analysis may give an aid in understanding subtle changes of pain processing under laser stimuli in migraine patients.

Written informed consent to publication was obtained from the patient(s).

